# Machine learning-based prediction of conversion coefficients for I-123 metaiodobenzylguanidine heart-to-mediastinum ratio

**DOI:** 10.1007/s12350-023-03198-3

**Published:** 2023-02-05

**Authors:** Koichi Okuda, Kenichi Nakajima, Chiemi Kitamura, Michael Ljungberg, Tetsuo Hosoya, Yumiko Kirihara, Mitsumasa Hashimoto

**Affiliations:** 1 Department of Physics, Kanazawa Medical University, 1-1 Daigaku, Uchinada, Kahoku, Ishikawa 920-0293 Japan; 2 Department of Functional Imaging and Artificial Intelligence, Graduate School of Advanced Preventive Medical Sciences, Kanazawa University, Kanazawa, Ishikawa Japan; 3 PDRadiopharma Inc., Kyobashi, Tokyo, Japan; 4 Medical Radiation Physics, Lund University, Lund, Scania Sweden; 5 Department of Radiation Science, Hirosaki University Graduate School of Health Sciences, Hirosaki-shi, Aomori, Japan

**Keywords:** ^123^I-MIBG, heart-to-mediastinum ratio, Monte Carlo simulation, collimator

## Abstract

**Purpose:**

We developed a method of standardizing the heart-to-mediastinal ratio in ^123^I-labeled *meta*-iodobenzylguanidine (MIBG) images using a conversion coefficient derived from a dedicated phantom. This study aimed to create a machine-learning (ML) model to estimate conversion coefficients without using a phantom.

**Methods:**

210 Monte Carlo (MC) simulations of ^123^I-MIBG images to obtain conversion coefficients using collimators that differed in terms of hole diameter, septal thickness, and length. Simulated conversion coefficients and collimator parameters were prepared as training datasets, then a gradient-boosting ML was trained to estimate conversion coefficients from collimator parameters. Conversion coefficients derived by ML were compared with those that were MC simulated and experimentally derived from 613 phantom images.

**Results:**

Conversion coefficients were superior when estimated by ML compared with the classical multiple linear regression model (root mean square deviations: 0.021 and 0.059, respectively). The experimental, MC simulated, and ML-estimated conversion coefficients agreed, being, respectively, 0.54, 0.55, and 0.55 for the low-; 0.74, 0.70, and 0.72 for the low-middle; and 0.88, 0.88, and 0.88 for the medium-energy collimators.

**Conclusions:**

The ML model estimated conversion coefficients without the need for phantom experiments. This means that conversion coefficients were comparable when estimated based on collimator parameters and on experiments.

**Supplementary Information:**

The online version of this article contains supplementary material available 10.1007/s12350-023-03198-3.

## Background

Cardiac scintigraphy with the noradrenaline analog ^123^I-labeled *meta*-iodobenzylguanidine (MIBG) is now an established means of evaluating heart failure^1-6^ and neurodegenerative diseases.^7-13^ The heart-to-mediastinum ratio (HMR) is calculated as the average count ratio of ^123^I-MIBG-derived activity in a cardiac region of interest (ROI) and that in the mediastinal ROI,^14,15^ can quantify cardiac sympathetic nerve activity in ^123^I-MIBG images.

The HMR considerably varies due to differences in the characteristics of gamma cameras and low-energy (LE), low-middle-energy (LME), and middle-energy (ME) collimators, that are used for ^123^I-MIBG imaging. High-energy photons emitted by ^123^I radionuclides can easily penetrate the thin septa of collimators. To overcome HMR variations due to collimator characteristics in gamma camera imaging systems, we developed an acrylic chest phantom that mimics the ^123^I-MIBG distribution in organs on planar images, then calibrated the HMR based on conversion coefficients.^16-22^ This method allowed for HMR values derived from various collimators and gamma camera combinations to be adjusted and unified to a standard gamma camera with a standard collimator. The method has been validated by phantom imaging at several medical centres in Europe and Japan.^17,23-26^ We then fit the conversion coefficients to the characteristics of collimators in ^123^I-MIBG imaging based on those results. However, the combinations of commercially available collimators and gamma cameras are numerous in current camera systems and as new cameras or collimators become available, further phantom imaging will be needed.

We, therefore, propose a new standardization method that does not require phantom experiments. We aimed to simulate images of ^123^I-MIBG phantoms using the Monte Carlo (MC) method with various collimator parameters, to calculate conversion coefficients from simulated phantom images, and to generate a machine learning (ML) model that could estimate conversion coefficients from collimator parameters.

## Methods

### ***Calibration phantom for planar ***^***123***^***I-MIBG imaging***

**Figure 1:**
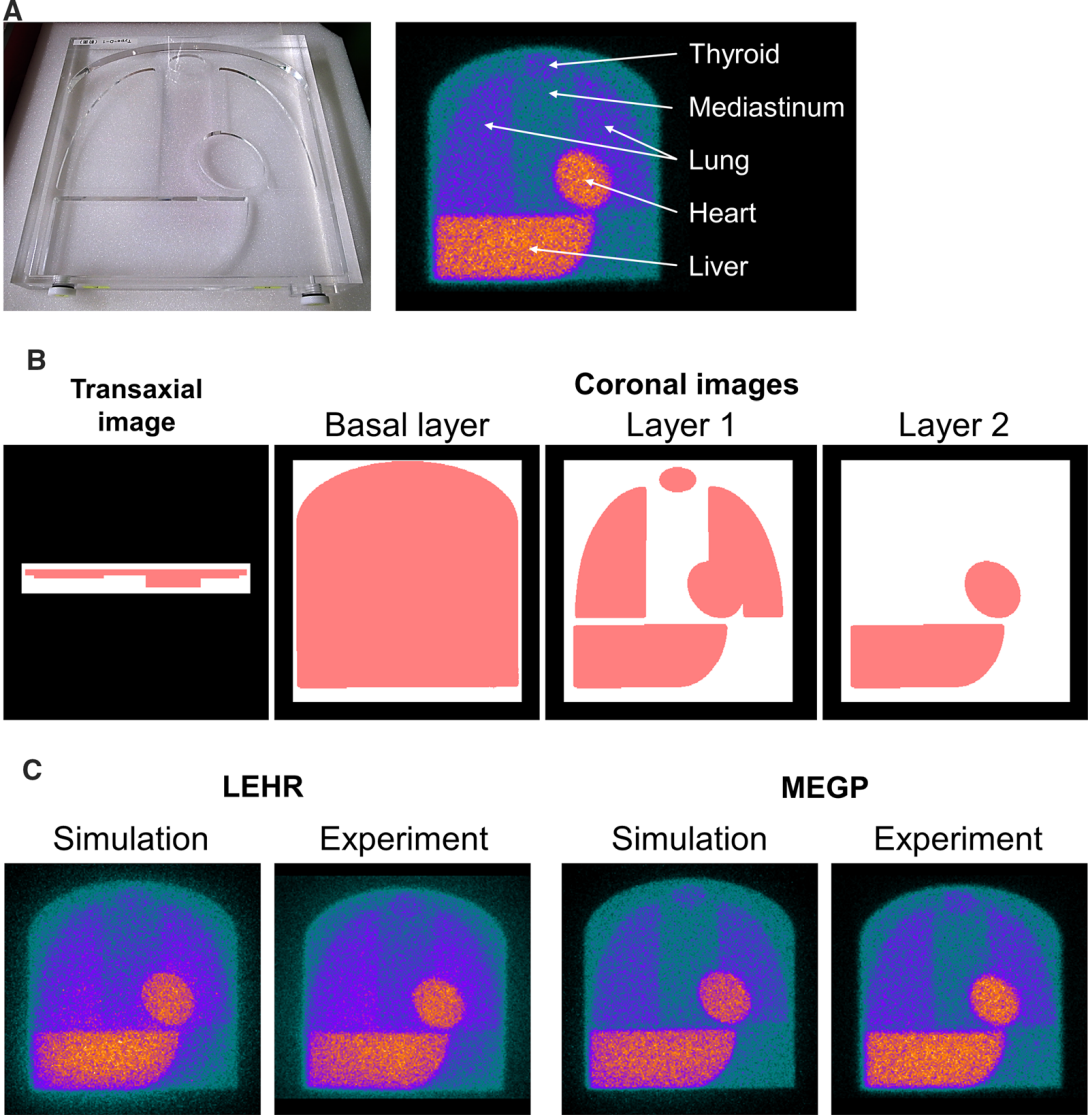
Monte Carlo **(**MC) simulation using ^123^I-MIBG phantom. **a** Acrylic ^123^I-MIBG phantom (left) and scintigraphic planar image (right). **b** Digital phantom for MC simulation. Density (white) and activity (red) maps of X-ray CT images acquired from acrylic phantom. (**c**) Simulation and experimental images with LEHR and MEGP collimators

A flat, polymethyl methacrylate phantom (width × depth × height: 380 × 380 × 50 mm^3^, Taisei Medical, Co. Ltd, Osaka, Japan) was developed to simulate ^123^I-MIBG distribution in the heart, mediastinum, liver, lungs, and thyroid gland (Figure 1a). A digital version of the phantom was created from an X-ray CT scan with different segmented compartments (Figure 1b) to obtain a three-layer map of activity. Background activity was represented in the basal layer. Cardiac, pulmonary, hepatic, and thyroid activities were represented in layers 1 and 2. Details of the phantom design have been published elsewhere.^16,22^

### ***Monte Carlo simulation for ***^***123***^***I-MIBG imaging***

We simulated ^123^I-MIBG planar imaging using the Simulated Imaging Nuclear Detectors (SIMIND) MC code.^27^ Since many simulation parameters for the gamma cameras were not available, we applied a standard gamma camera in all MC simulations that had a crystal thickness, width, and length, 9.5 (3/8 inch), 269.5, and 250 mm, respectively; backscatter material and cover thickness, 76 and 1.0 mm, respectively; energy resolution, 9.8% (FWHM at 140 keV); intrinsic resolution, 3.8 mm; energy window,159 keV ± 10.0%; matrix and pixel sizes, 256 × 256 and 2.21 mm, respectively. All MC simulations generated ^123^I-MIBG planar images using a total of 1.78 × 10^9^ photons.

*Simulation 1*: Eighteen collimators were simulated for the following gamma camera systems: Infinia, Millennium MG, and Millennium VG (GE Healthcare, Waukesha, WI, USA); PRISM-AXIS and IRIX (Picker Corporation, Cleveland, OH, USA); Brightview (Philips Medical Systems, Milpitas, CA, USA); Symbia and E.CAM (Siemens Healthineers, Erlangen, Germany). Table 1 shows details of the collimator parameters. All MC simulations were repeated three times per collimator.

**Table 1:** Collimator parameters in gamma camera systems

Gamma camera	Collimator	Hole diameter (mm)	Septum thickness (mm)	Length (mm)
GE				
Infinia	LEHR	1.50	0.20	35.00
	ELEGP	2.50	0.40	40.00
	MEGP	3.00	1.05	58.00
Millennium VG	LEHR	1.50	0.20	35.00
	LEGP	1.90	0.20	35.00
	MEGP	3.00	1.05	58.00
Millennium MG	LEHR	1.80	0.18	41.00
	LEGP	2.50	0.25	43.00
	MEGP	3.00	1.20	42.00
Philips				
Brightview	CHR	2.03	0.152	48.00
	MEGP	3.40	0.86	58.40
SIEMENS				
E.CAM	LEHR	1.11	0.16	24.05
	MELP	2.94	1.14	40.64
Symbia	LEHR	1.11	0.16	24.05
	MELP	2.94	1.14	40.64
Picker				
AXIS/IRIX	LEHR	1.22	0.20	27.00
	LEGP	1.40	0.25	25.40
	MEGP	3.40	0.86	58.40

*CHR*, cardiac high-resolution; *ELEGP*, extended low-energy general-purpose; *LEAP*, low-energy all-purpose; *LEGP*, low-energy general-purpose; *LEHR*, low-energy high-resolution; *LMEGP*, low-medium-energy general-purpose; *MEGP*, medium-energy general-purpose; *MELP*, medium-energy low-penetration

*Simulation 2*: We simulated 283 collimators with hole diameters of 1.0, 1.25, 1.50, 1.75, 2.0, 2.5, 3.0, and 3.5 mm; septal thickness of 0.15, 0.2, 0.25, 0.3, 0.4, 0.5, 0.6, 0.7, 0.8, 0.9, 1.2, and 1.5 mm; and lengths of 20, 25, 30, 35, 40, 45, 50, 55, and 60 mm.

*Simulation 3*: Three collimators were simulated for the Discovery NM series (GE Healthcare) with respective to hole diameters, septal thicknesses, and collimator lengths of 1.5, 0.2, and 35.0 mm, for the low-energy high-resolution (LEHR) type; 2.5, 0.4, and 40.0 mm for the extended low-energy general-purpose (ELEGP) type; 3.0, 1.05, and 58.0 mm for the medium-energy general-purpose (MEGP) type. All MC simulations were repeated five times per collimator.

### ^***123***^***I-MIBG phantom image database for validating results of MC simulation***

We generated a multicenter database of ^123^I-MIBG experimental phantom images accumulated in Japan over a period of 15 years.^16^ Two image datasets were selected from the database to validate the results of MC simulations 1 and 3 as follows.

*Validation dataset for MC simulation 1*: Seven gamma cameras and collimators were selected from the database along with 153, 33, 40, 36, 73, 39, and 60 phantom images acquired using Infinia, Millennium MG, Millennium VG, BrightView, AXIS/IRIX, Symbia, and E.CAM systems, respectively.

*Validation dataset for MC simulation 3*: We selected phantom images from the database using the gamma camera of Discovery NM 630, 640, 670, 830, and 850 systems (GE Healthcare). Among these, 26, 107, and 46 were acquired using LEHR, ELEGP, and MEGP collimators.

Planar images in both datasets were acquired from a phantom containing 111 MBq of ^123^I-MIBG. Planar imaging proceeded with 256 × 256 or 512 × 512 matrices. The photopeak window of ^123^I was centered at 159 keV with a 20% energy window. The maximum, minimum, and median acquisition durations were 600, 180, and 300 s, respectively.

### Monte Carlo simulation using standard and original detector conditions

We confirmed the conversion coefficients derived from standard detector conditions with those from original detector conditions in six commercially available gamma cameras. Table 2 shows the crystal thickness, intrinsic resolution, and energy resolution in the original detector conditions. The other detector parameters for backscatter material and cover thickness were set as 76 mm and 1.0 mm, respectively. Regarding the imaging condition, the energy window, matrix size, and pixel sizes were set as 159 keV ± 10.0%, 256 × 256, and 2.21 mm, respectively. All MC simulations generated ^123^I-MIBG planar images using a total of 1.78 × 109 photons. All MC simulations were repeated five times in the standard and original detector conditions.

**Table 2:** Conversion coefficients derived with original and standard detector conditions

Gamma camera	Intrinsic resolution (mm)	Energy resolution (%)	Collimator	Conversion coefficients		*p* value
				Original detector condition	Standard detector condition	
GE						
Infinia	3.8	9.8	LEHR	0.554 ± 0.013	0.549 ± 0.011	n.s
(3/8-inch crystal)			MEGP	0.893 ± 0.012	0.889 ± 0.012	n.s
Millennium MG	3.7	9.7	LEHR	0.536 ± 0.004	0.533 ± 0.016	n.s
(3/8-inch crystal)			MEGP	0.874 ± 0.006	0.866 ± 0.013	n.s
NMCT	3.7	9.5	LEHR	0.547 ± 0.015	0.544 ± 0.011	n.s
(3/8-inch crystal)			MEGP	0.878 ± 0.013	0.881 ± 0.010	n.s
NMCT	4.5	9.5	LEHR	0.588 ± 0.009	0.589 ± 0.016	n.s
(5/8-inch crystal)			MEGP	0.906 ± 0.007	0.905 ± 0.010	n.s
Philips						
Brightview	3.3	9.6	CHR	0.489 ± 0.004	0.489 ± 0.005	n.s
(3/8-inch crystal)			MEGP	0.853 ± 0.009	0.853 ± 0.019	n.s
SIEMENS						
E.CAM	3.8	9.9	LEHR	0. 555 ± 0.004	0.552 ± 0.006	n.s
(3/8-inch crystal)			MEGP	0.860 ± 0.016	0.869 ± 0.006	n.s
E.CAM	4.5	9.9	LEHR	0.606 ± 0.014	0.611 ± 0.007	n.s
(5/8-inch crystal)			MEGP	0.883 ± 0.010	0.872 ± 0.008	n.s
Picker						
AXIS/IRIX	3.3	9.5	LEHR	0.558 ± 0.009	0.565 ± 0.010	n.s
(3/8-inch crystal)			MEGP	0.865 ± 0.011	0.863 ± 0.008	n.s

The intrinsic resolution of 3.8 mm and energy resolution of 9.8% (FWHM at 140 keV) were used in the standard detector condition. *CHR*, cardiac high-resolution; *LEHR*, low-energy high-resolution; *MEGP*, medium-energy general-purpose

### Image analysis

A calibration factor was calculated from two HMR values derived from anterior (HMR_Ant_) and posterior (HMR_Post_) planar ^123^I-MIBG phantom images as a conversion coefficient:

$$ {\text{Conversion}}\;{\text{coefficient}}\; = \;\left\{ {\left( {{\text{HMR}}_{{{\text{Ant}}}} \; + \;{\text{HMR}}_{{{\text{Post}}}} } \right)/2\; - \;1} \right\}/\left\{ {\left( {2.6 \, + \, 3.5} \right)/2 \, - 1} \right\}, $$

where 2.6 and 3.5 are the respective designated HMRs in the anterior and posterior views of the calibration phantom.^22^ We developed ROI setting software for phantom image analysis. Standardized circular and rectangular ROIs were automatically delineated on the heart and mediastinum. These ROI sizes and positioning were determined based on the smartMIBG software program.^28^

### Machine learning (ML) model

We estimated conversion coefficients using an ML algorithm for regression analysis. We applied a gradient-boosting regression algorithm using scikit-learn^29^ to estimate conversion coefficients by building seven models with hole diameter, septal thickness, and collimator length. The conversion coefficients and related collimator parameters were randomly divided into training and validation datasets in a ratio of 3:2. We performed cross-validation for the machine learning prediction models using a fourfold model. The optimal ranges of hyperparameter values for the ML algorithm of 0.01-0.5 for the learning rate, 1-100 for the number of estimators, and 1-8 for the maximum depth of the individual regression estimators were determined using a grid search. Default values were applied to the remaining hyperparameters. Feature importances of the hole diameter, septal thickness, and collimator length were evaluated using the permutation feature importance provided by the scikit-learn.^30^

### Conventional multiple linear regression model

We estimated conversion coefficients using a conventional multiple linear regression model. Collimator parameters of the hole diameter, septal thickness, and collimator length were used to build the model. The conversion coefficients and related collimator parameters were randomly divided into training and validation datasets in a ratio of 3:2. The software machine learning library, scikit-learn,^29^ was used.

### Statistical analysis

All continuous parametric variables are expressed as means ± standard deviation (SD), and non-parametric data are presented as medians. Differences in continuous variables were analyzed using Student t-tests and Wilcoxon signed-rank tests. Multiple comparisons of continuous variables were assessed using Tukey–Kramer tests. Differences in paired continuous data were analyzed using paired t-tests. A root mean square error (RMSE) is used to measure the error of a model in predicting conversion coefficients. All statistical tests were two-tailed, and values with *p* < 0.05 were considered significant. All data were statistically analyzed using JMP version 11.2.1 (SAS Institute Inc., Cary, NC, USA).

## Results

**Figure 2:**
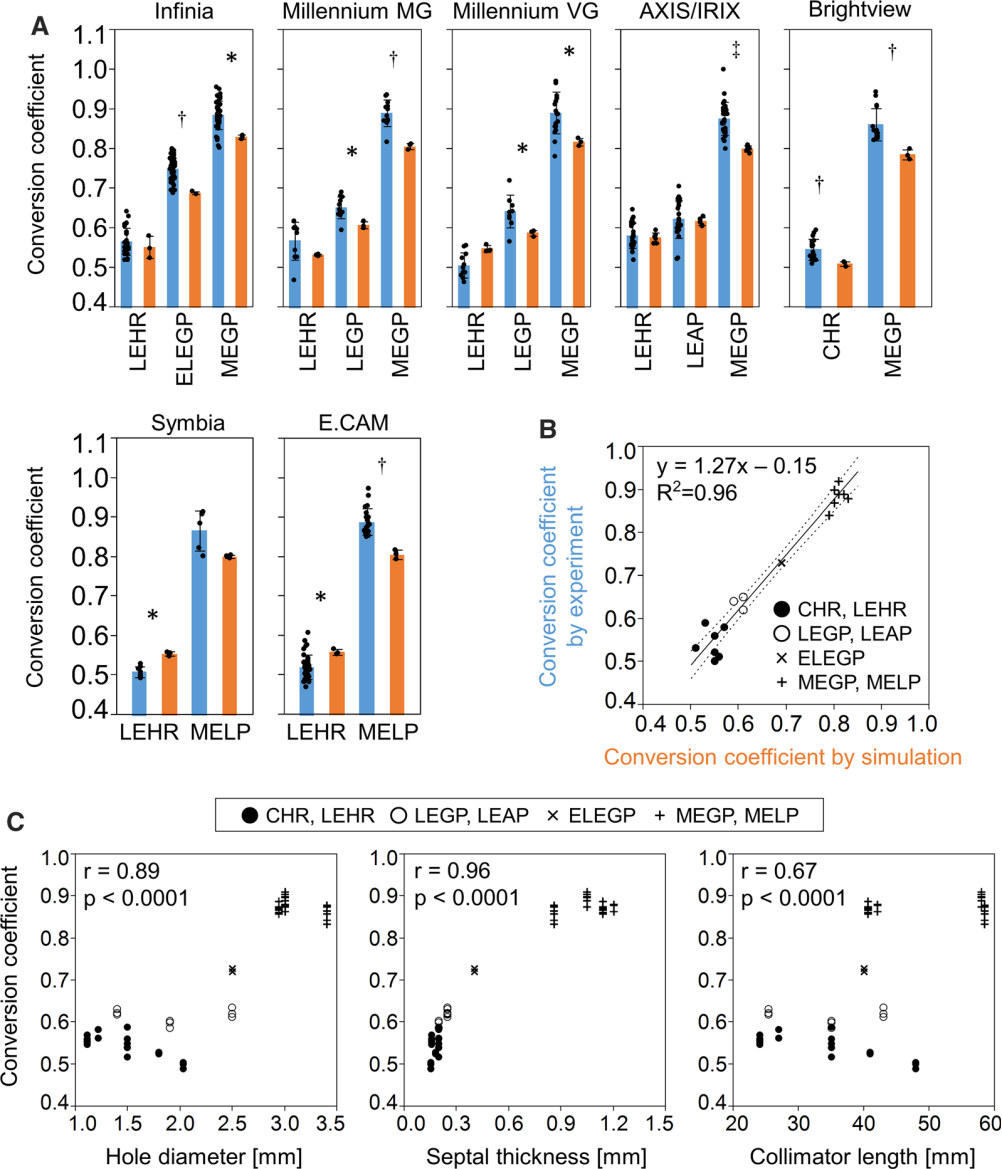
Conversion coefficients derived from Monte Carlo simulated ^123^I-MIBG phantom images. **a** Averaged experimental (blue) and simulated (orange) conversion coefficients in seven gamma camera systems. *p < 0.05, ^†^*p* < 0.01, ^‡^*p* < 0.001. **b** Regression line of experimental *vs.* simulated conversion coefficients. **c** Correlations between conversion coefficients and hole diameter (left), septal thickness (center), and length (right)

The quality of the simulated and experimental phantom images was equivalent in terms of LEHR and MEGP collimator conditions (Figure 1c). A comparison of the average of simulation and experimental conversion coefficients among seven camera systems revealed that higher mean values were in the experimental than the simulated images for 10 collimators (Figure 2a). The regression line of averaged experimental conversion coefficients (Y) as a function of simulated values (X) was Y = 1.27X-0.15 (R^2^ = 0.96; Figure 2b). The simulated conversion coefficients were compensated using this equation in subsequent analyses. Hole diameter and septal thickness closely correlated with conversion coefficients (Figure 2c).

When the original detector parameters: the intrinsic resolutions were 3.3-3.8 mm for the 3/8-inch crystal and 4.5 mm for the 5/8-inch crystal; and the energy resolution were 9.5-9.9%, were used in MC simulation, the conversion coefficients derived from the original and standard detector conditions were comparable in LE and ME collimators (Table 2). The influence of the differences between original and standard detector parameters on conversion coefficients was small. A lookup table of conversion coefficients is available in Table 2. The conversion coefficients of LE collimators were within the ranges of 0.49-0.56 for the 3/8-inch crystal and of 0.59-0.61 for the 5/8-inch crystal. The conversion coefficients of ME collimators were 0.85-0.89 and 0.88-0.91 in the 3/8-inch and 5/8-inch crystals, respectively.

**Figure 3:**
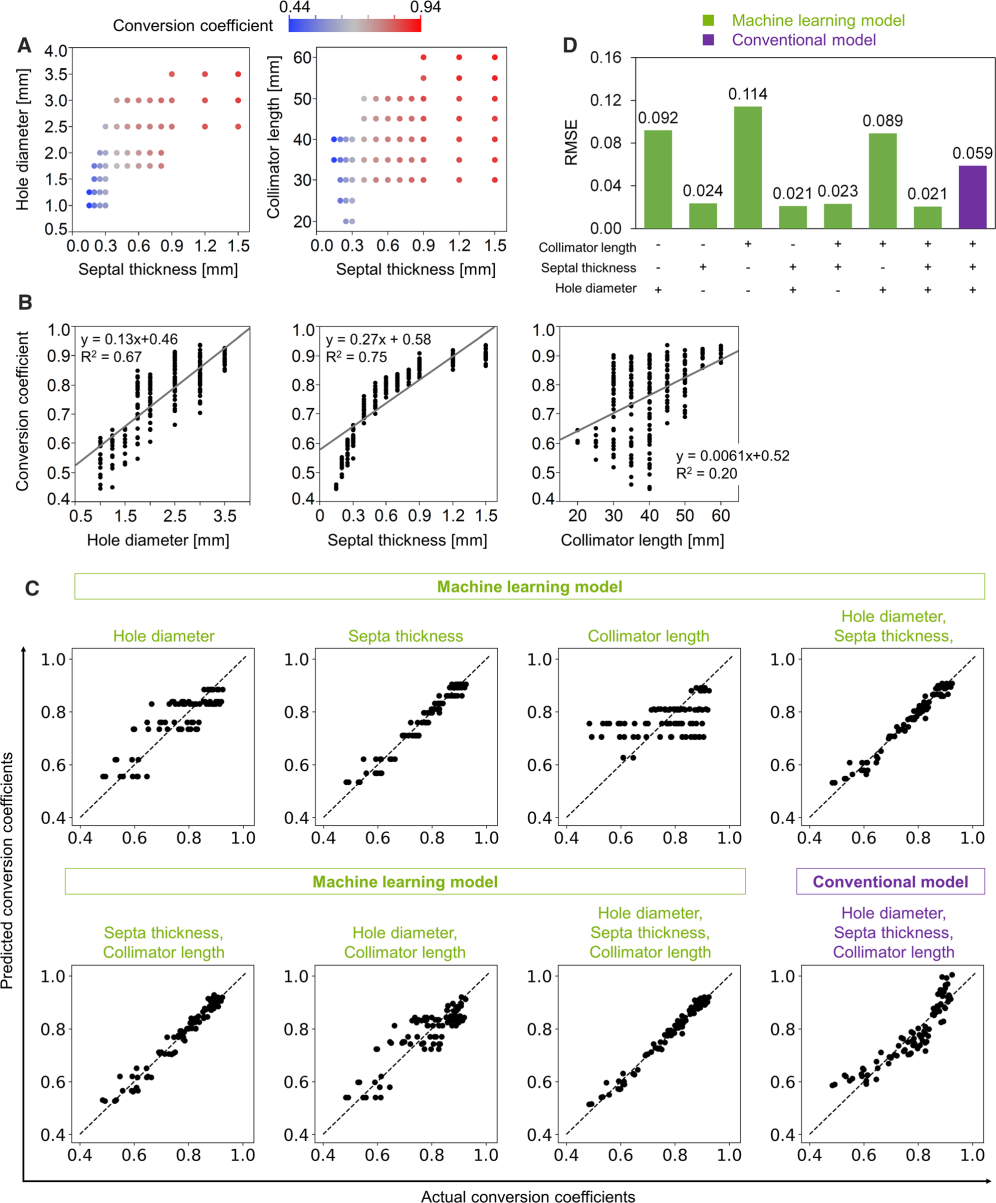
Machine learning (ML) estimation of conversion coefficients using collimator parameters. **a** Conversion coefficients were calculated using 210 collimator parameters. **b** Relationships between conversion coefficients and collimator parameters: hole diameter (left), septal thickness (center), and length (right). **c** Agreement between actual and predicted conversion coefficients in seven ML and conventional multiple linear regression models. **d** RMSE values between actual and predicted conversion coefficients in ML and conventional models

The HMR and related conversion coefficients were calculated using 283 collimator parameters (Supplementary Figure 1). We excluded 73 from the following analyses due to high septal penetration (> 1.0%). Conversion coefficients calculated from the remaining 210 collimator parameters were all within the range of 0.44-0.94 (Figure 3a). Although correlations between conversion coefficients and collimator parameters were positive, the range of R^2^ values was 0.2-0.75 (Figure 3b). Agreements between actual and predicted conversion coefficients were evaluated using scatter plots (Figure 3c). Analysis of the seven ML models using combinations of collimator diameter, septal thickness, and collimator length, revealed that the smallest RMSE was 0.0207 when these collimator parameters were incorporated (Figure 3d). Tuned hyperparameters for this ML condition were as follows: learning rate, 0.33; the number of estimators, 25; maximum depth of individual regression estimators, two. Conventional multiple linear regression analysis using the three parameters provided an RMSE of 0.0588. The higher score of feature importance was 1.5 ± 0.15 for septal thickness and the lower scores were 0.057 ± 0.0088 for hole diameter and 0.0080 ± 0.0017 for collimator length.

**Figure 4:**
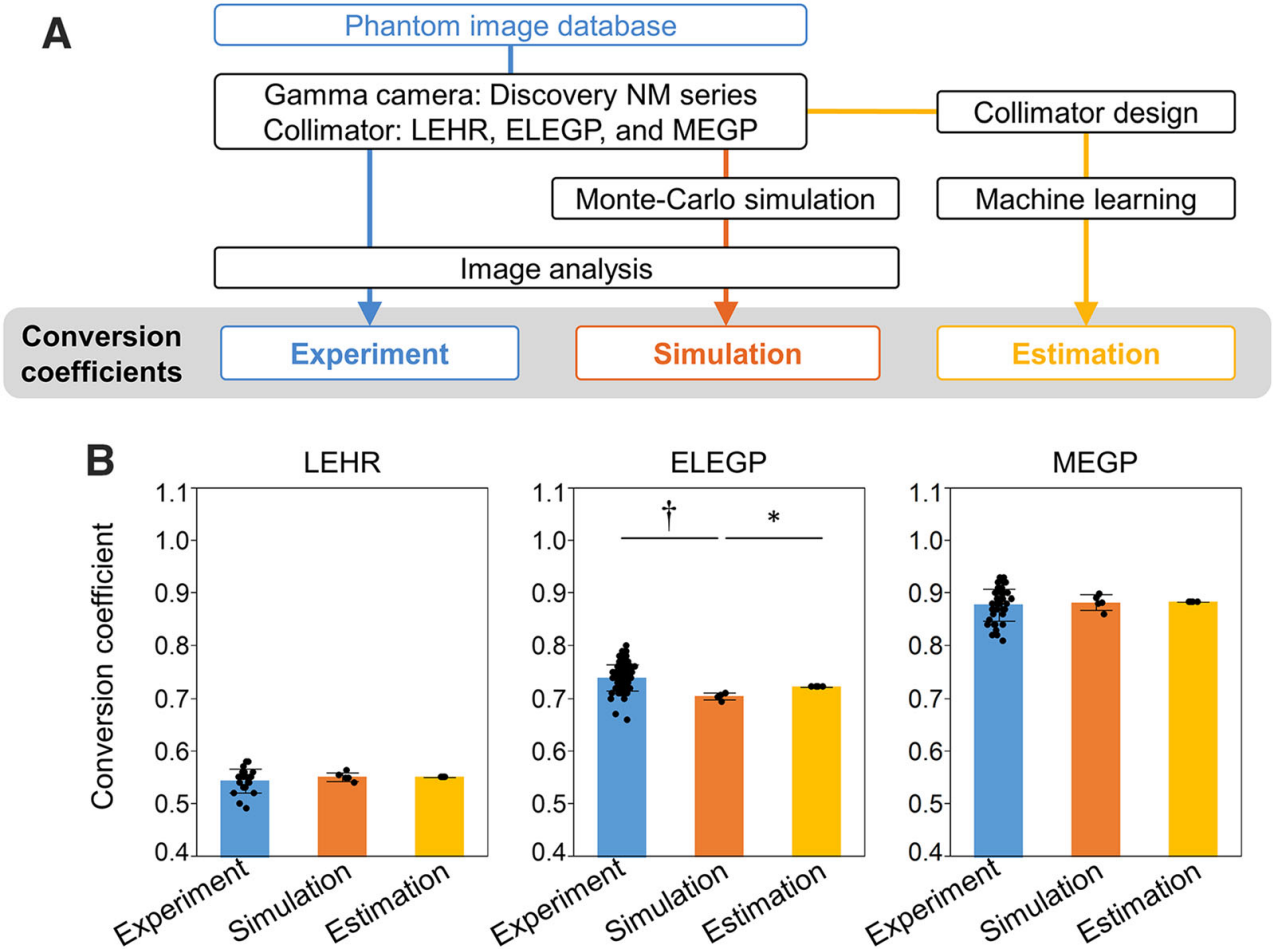
Comparisons of experimental, MC simulated, and ML-estimated conversion coefficients. **a** Experimental, simulated, and estimated conversion coefficients were calculated for LEHR, ELEGP, and MEGP collimators. **b** Average of experimental (blue), MC simulated (orange), and ML-estimated (yellow) conversion coefficients for three collimators. *p < 0.05, ^†^*p* < 0.01

The experimentally derived, MC simulation, and ML-estimated conversion coefficients in ^123^I-MIBG phantom images (Figure 4a) were, respectively, 0.54 ± 0.023, 0.55 ± 0.0082, and 0.55 for LEHR, 0.74 ± 0.025, 0.70 ± 0.0066, and 0.72 for ELEGP, and 0.88 ± 0.030, 0.88 ± 0.014, and 0.88 for MEGP (Figure 4b).

## Discussion

The main findings of the present study were that conversion coefficients were slightly underestimated when simulated compared with experimental results, and were corrected with the regression line of experimental conversion coefficients on simulated results. Simulated conversion coefficients closely were correlated with collimator parameters. The ML models revealed that conversion coefficients could be precisely estimated from collimator parameters. The experimentally derived and ML-estimated conversion coefficients closely agreed in the validation study.

We have promoted a phantom-based methodology to standardize the HMR in ^123^I-MIBG imaging. Many cardiological and neurological studies have determined HMRs without standardization^1-3,7,10,31,32^ and this has led to wide variations in clinical normal values and HMR thresholds. Clinical criteria to determine the prognosis of heart failure would change by these HMR values as the optimal threshold of the HMR for diagnosing neurological disorders would be influenced by non-standardized conditions. Moreover, regardless of whether diagnostic models were generated using statistical or ML approaches based on non-standardized HMRs, variations in these values cannot be excluded from estimations. Therefore, a standard HMR should be mandatory in ^123^I-MIBG imaging.

The simulated conversion coefficients were underestimated compared with those determined under actual MEGP collimator conditions. Assessment of the penetration fraction of the ME collimator in published simulation studies revealed that Rault et al. found a 3% penetration fraction using a point source.^33^ Moreover, Konik et al. found ~ 4% penetration fraction using a point source and the 4D extended cardiac-torso (XCAT) digital phantom.^34^ The geometry and tracking 4 (GEANT4) application for tomographic emission (GATE) package^35^ was used in these studies. The mean penetration fraction was 7.2% in our simulation. Slight differences in the penetration fraction could have influenced the simulated conversion coefficients.

Numerous parameters for gamma cameras and collimators should be fixed to reproduce actual phantom experiments in MC simulations. When the densities of the collimator material were fixed at 11.34 (Pb,100%); 11.25 (Pb 98%, Sb2%); 11.20 (Pb 97%, Sb 3%); 11.15 (Pb 96%, Sb 4%); and 11.06 (Pb 94%, Sb 6%) g/cm^3^, the conversion coefficients changed from 0.80 to 0.83 even with the ME collimator. The contribution of high-energy photon backscatter from the photomultiplier tube was also fixed. When the backscatter material thickness was 3.8, 7.6 (default), and 11.4 cm, the conversion coefficients slightly changed from 0.79 to 0.81. In summary, the effects of collimator material densities and backscatter material thickness on the conversion factor were limited under the conditions of MC simulation.

Some factors influence the conversion coefficients except collimators and cameras. We exhibited that the primary energy window setting for the image acquisition condition affected the conversion coefficients.^16^ The conversion coefficients showed higher values when images were acquired with the energy windows of 159 keV ± 7.5% than 159 keV ± 10%. However, the energy window of 159 keV ± 7.5% was not popular as ^123^I-MIBG acquisition condition. Conversely, equivalent conversion coefficients were observed in imaging matrices of 256 and 512.^16^ When we evaluated the variations of conversion coefficients in two dual-head gamma camera positioning: parallel-mode and L-mode configurations, the conversion coefficients did not differ significantly between the two configurations. In addition, even when the distances between phantom and the opposite gamma camera that did not acquire planar data were changed, the conversion coefficients were stable at any distance. The effects of the gamma camera configurations on the variation of conversion coefficients may be therefore negligible.

The ML model provided reasonable conversion coefficients using only the specifications of collimator designs. Since the collimator parameters were nonlinearly associated with conversion coefficients, ML would be suitable for these data compared with simple statistical and multiple linear regression models. Considering the selection of parameters in collimator design, septal thickness was pivotal to estimate conversion coefficients. Moreover, the combination of septal thickness, hole diameter, and collimator length generated excellent estimates of conversion coefficients.

The interpretation of the ML model is important for applying the ML to simulation data. We have preliminary compared gradient-boosting regression with kNN, linear regression, and support vector machine using the orange data mining toolbox in python^36^ (Supplementary Figure 2). A total of 210 conversion coefficients with three collimator parameters were used as test data. A cross-validation using a fivefold model was applied to calculate RMSE values. The gradient-boosting regression showed the smallest RMSE of 0.016. The gradient-boosting regression model was suitable for our dataset.

Although MC simulation can directly estimate conversion coefficients based on collimator designs, MC simulations of ^123^I-MIBG phantom imaging are very laborious. Consequently, we estimated conversion coefficients using the ML algorithm instead of MC simulation. However, we suggest that actual phantom experiences at each institution are still needed to obtain conversion coefficients, particularly as new gamma cameras and collimators are developed. The vendors of the cameras may sometimes update and modify collimator designs. When the collimators will be modified or newly created, we need to perform MIBG phantom experiments to calculate conversion coefficients fitted for the new specifications. Our results are, however, utilized for Anger-type gamma cameras with lead collimators. When a new collimator is available with its specifications, our ML model might be able to estimate appropriate conversion coefficients for the modified collimator even without phantom experiments. In addition, the applicability of current cadmium-zinc-telluride detectors awaits validation. When calculated conversion coefficients are out of range in institutional phantom images, the ML model can provide a standard conversion coefficient based on combinations of collimator and gamma camera parameters.

Our study has several limitations. MC simulations cannot reproduce all the physical phenomena involved in ^123^I-MIBG imaging. Further, MC simulations with numerous collimator parameters are needed to improve the performance of the ML model. The clinical impact on patient diagnosis was not evaluated using the ML-estimated conversion coefficients in this study. We evaluated the clinical impact on patient diagnosis using conversion coefficients derived from real phantom images in our previous study.^16^ The net reclassification improvement in 21 normal subjects and 12 patients with heart failure showed 14.3% when the HMR values were corrected using the conversion coefficients. Therefore, if we can assume that the conversion coefficients by the simulation and the phantom experiment were equivalent, comparable clinical impacts would be provided by the simulations and experiments.

## New knowledge gained

The MC simulations were very helpful for estimating the results of basic phantom experiments in ^123^I-MIBG images. These results could serve as training and validation materials for the ML algorithm. In addition to actual ^123^I-MIBG phantom experiments, combined ML and MC simulations would aid HMR standardization.

## Conclusions

We aimed to estimate conversion coefficients for the HMR ratio in ^123^I-MIBG images using ML models. We found that the ML model could reasonably estimate conversion coefficients based on the fundamental collimator parameters of septal thickness, hole length, and diameter. Conversion coefficients in the validation study were equivalent between the ML model and the experimental results. Our proposed methodology will contribute to the HMR standardization of ^123^I-MIBG scintigraphy.

## Supplementary Information

Below is the link to the electronic supplementary material.

Electronic supplementary material 1 (PDF 43 kb)

Electronic supplementary material 2 (PPTX 1504 kb)

## Abbreviations

^123^I-MIBG: ^123^I-labeled *meta*-iodobenzylguanidine
HMR: Heart-to-mediastinum count ratio
LE: Low energy
MC: Monte Carlo
ME: Medium energy
ML: Machine learning
