# Supplementary material for: Machine learning-based prediction of conversion coefficients for I-123 metaiodobenzylguanidine heart-to-mediastinum ratio
Source: J Nucl Cardiol. 2023 Feb 5;30(4):1630–41. doi: 10.1007/s12350-023-03198-3 (PMC10372132; doi:10.1007/s12350-023-03198-3)
Supplement: Supplementary file 1 — Electronic supplementary material 1 (PDF 43 kb) [file 12350_2023_3198_MOESM1_ESM.pdf]

Heart to mediastinum ratio

Diameter	Septa	Length 20	25	30	35	40	45	50	55	60
1.0	0.15	2.0	1.9	1.9	1.9	1.8				
	0.20	2.0	1.9	1.9	1.8	1.9				
	0.25	2.0	2.0	1.9	1.9	2.0				
	0.30	2.0	2.0	2.0	1.9	2.0				
1.25	0.15	2.1	2.0	1.9	1.9	1.8				
	0.20	2.1	2.0	1.9	1.9	1.9				
	0.25	2.1	2.0	2.0	2.0	1.9				
	0.30	2.1	2.0	2.0	2.0	2.0				
1.50	0.15	2.2	2.1	2.0	1.9	1.9				
	0.20	2.1	2.0	2.0	1.9	1.9				
	0.25	2.1	2.1	2.1	2.0	2.0				
	0.30	2.1	2.1	2.1	2.0	2.0				
1.75	0.15	2.2	2.2	2.0	1.9	1.9				
	0.20	2.2	2.1	2.1	1.9	1.9				
	0.25	2.2	2.1	2.1	2.0	2.0				
	0.30	2.2	2.2	2.1	2.0	2.0				
	0.40			2.1	2.2	2.1	2.2	2.2		
	0.50			2.2	2.2	2.2	2.2	2.2		
	0.60			2.2	2.3	2.3	2.3	2.3		
	0.70			2.3	2.3	2.3	2.3	2.3		
	0.80			2.3	2.3	2.3	2.3	2.4		
2.0	0.15	2.3	2.2	2.1	2.0	1.9				
	0.20	2.3	2.2	2.1	2.0	2.0				
	0.25	2.2	2.2	2.1	2.1	2.0				
	0.30	2.2	2.2	2.1	2.1	2.1				
	0.40			2.2	2.1	2.2	2.1	2.2		
	0.50			2.2	2.2	2.2	2.2	2.2		
	0.60			2.3	2.2	2.2	2.3	2.3		
	0.70			2.3	2.3	2.3	2.2	2.3		
	0.80			2.3	2.3	2.3	2.3	2.3		
2.5	0.15	2.3	2.3	2.2	2.1	2.0				
	0.20	2.3	2.2	2.2	2.1	2.0				
	0.25	2.3	2.2	2.2	2.1	2.1				
	0.30	2.3	2.2	2.2	2.1	2.1				
	0.40			2.2	2.2	2.2	2.1	2.2		
	0.50			2.3	2.3	2.3	2.2	2.2		
	0.60			2.3	2.3	2.3	2.2	2.3		
	0.70			2.3	2.3	2.3	2.3	2.3		
	0.80			2.3	2.3	2.3	2.3	2.3		
	0.90			2.3	2.4	2.3	2.4	2.4	2.4	2.4
	1.20			2.4	2.4	2.4	2.4	2.4	2.4	2.4
	1.50			2.4	2.4	2.4	2.4	2.4	2.5	2.4
3.0	0.40			2.3	2.2	2.2	2.2	2.2		
	0.50			2.3	2.3	2.3	2.3	2.3		
	0.60			2.3	2.3	2.3	2.3	2.3		
	0.70			2.3	2.3	2.3	2.3	2.3		
	0.80			2.3	2.3	2.3	2.3	2.3		
	0.90			2.3	2.4	2.4	2.3	2.4	2.4	2.4
	1.20			2.4	2.4	2.4	2.4	2.5	2.5	2.4
	1.50			2.4	2.4	2.4	2.4	2.5	2.4	2.4
3.5	0.90			2.4	2.3	2.4	2.4	2.4	2.4	2.5
	1.20			2.4	2.4	2.4	2.4	2.4	2.4	2.4
	1.50			2.4	2.4	2.4	2.4	2.4	2.4	2.5

Color scale: 1.78 – 2.48

Conversion coefficient

Diameter	Septa	Length 20	25	30	35	40	45	50	55	60
1.0	0.15	0.60	0.52	0.47	0.46	0.44				
	0.20	0.58	0.54	0.52	0.48	0.49				
	0.25	0.60	0.59	0.56	0.53	0.55				
	0.30	0.61	0.60	0.59	0.55	0.62				
1.25	0.15	0.68	0.59	0.52	0.50	0.45				
	0.20	0.64	0.58	0.55	0.52	0.51				
	0.25	0.65	0.61	0.58	0.56	0.52				
	0.30	0.64	0.63	0.60	0.59	0.61				
1.50	0.15	0.76	0.65	0.57	0.52	0.48				
	0.20	0.70	0.62	0.56	0.53	0.53				
	0.25	0.69	0.64	0.61	0.59	0.56				
	0.30	0.70	0.65	0.64	0.60	0.63				
1.75	0.15	0.80	0.72	0.64	0.57	0.54				
	0.20	0.75	0.68	0.62	0.57	0.55				
	0.25	0.75	0.68	0.63	0.60	0.60				
	0.30	0.74	0.69	0.65	0.62	0.62				
	0.40			0.69	0.70	0.69	0.72	0.69		
	0.50			0.73	0.73	0.70	0.72	0.76		
	0.60			0.77	0.77	0.79	0.83	0.79		
	0.70			0.79	0.80	0.79	0.81	0.81		
	0.80			0.81	0.80	0.82	0.83	0.85		
2.0	0.15	0.83	0.77	0.69	0.60	0.56				
	0.20	0.79	0.72	0.64	0.60	0.56				
	0.25	0.78	0.71	0.66	0.62	0.61				
	0.30	0.77	0.71	0.69	0.65	0.65				
	0.40			0.72	0.70	0.69	0.67	0.71		
	0.50			0.74	0.73	0.74	0.75	0.79		
	0.60			0.77	0.76	0.77	0.79	0.83		
	0.70			0.80	0.80	0.80	0.78	0.82		
	0.80			0.82	0.82	0.83	0.81	0.84		
2.5	0.15	0.86	0.81	0.76	0.69	0.63				
	0.20	0.85	0.79	0.73	0.66	0.61				
	0.25	0.82	0.76	0.70	0.68	0.64				
	0.30	0.81	0.76	0.72	0.68	0.66				
	0.40			0.75	0.73	0.72	0.71	0.73		
	0.50			0.78	0.80	0.79	0.76	0.78		
	0.60			0.80	0.81	0.78	0.78	0.81		
	0.70			0.81	0.80	0.83	0.82	0.82		
	0.80			0.82	0.81	0.84	0.85	0.85		
	0.90			0.82	0.86	0.84	0.86	0.88	0.89	0.89
	1.20			0.87	0.85	0.87	0.90	0.90	0.90	0.91
	1.50			0.87	0.89	0.89	0.91	0.87	0.91	0.89
3.0	0.40			0.78	0.76	0.74	0.74	0.70		
	0.50			0.81	0.78	0.79	0.78	0.77		
	0.60			0.81	0.80	0.81	0.80	0.82		
	0.70			0.82	0.83	0.82	0.83	0.84		
	0.80			0.84	0.83	0.83	0.83	0.85		
	0.90			0.83	0.86	0.86	0.85	0.88	0.86	0.88
	1.20			0.87	0.88	0.88	0.90	0.92	0.91	0.91
	1.50			0.89	0.90	0.90	0.94	0.90	0.91	0.93
3.5	0.90			0.86	0.85	0.87	0.87	0.88	0.87	0.91
	1.20			0.88	0.88	0.89	0.89	0.88	0.92	0.92
	1.50			0.90	0.89	0.90	0.89	0.90	0.93	0.93

Color scale: 0.44 – 0.94

Supplementary Figure 1. Heart-to-mediastinum ratios and conversion coefficients in MC simulation 2.

Septal penetration was > 1.0% under 73 simulation conditions (selected cells in heat maps). Therefore, these data were excluded from simulation results.

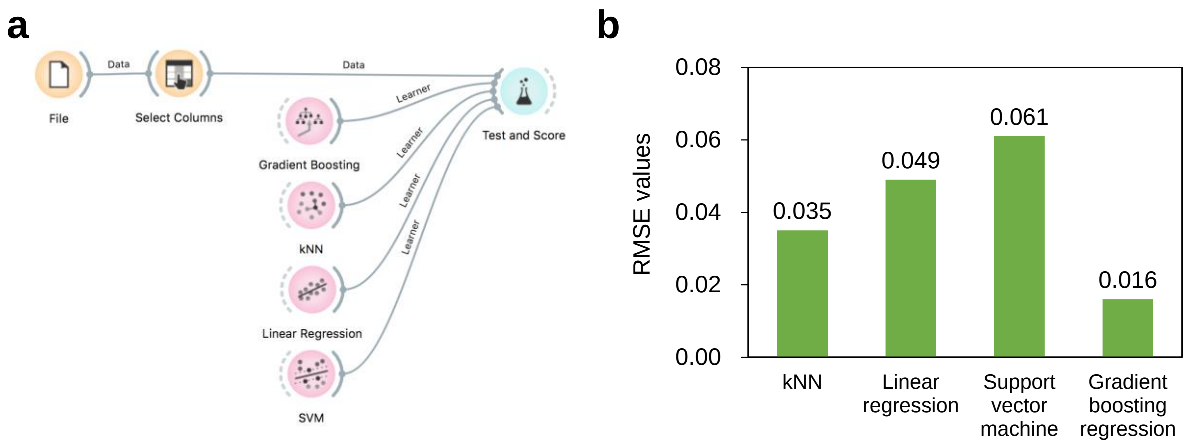

Supplementary Figure 2. The selection of ML models in the estimation of conversion coefficients.

a. The orange data mining toolbox in python was used for the selection of ML models: kNN, linear regression, support vector machine, and gradient boosting regression.

b. RMSE values between actual and predicted conversion coefficients in the four ML models.
