# Supplementary material for: Machine learning-based prediction of conversion coefficients for I-123 metaiodobenzylguanidine heart-to-mediastinum ratio
Source: J Nucl Cardiol. 2023 Feb 5;30(4):1630–41. doi: 10.1007/s12350-023-03198-3 (PMC10372132; doi:10.1007/s12350-023-03198-3)
Supplement: Supplementary file 2 — Electronic supplementary material 2 (PPTX 1504 kb) [file 12350_2023_3198_MOESM2_ESM.pptx]

## Slide 1
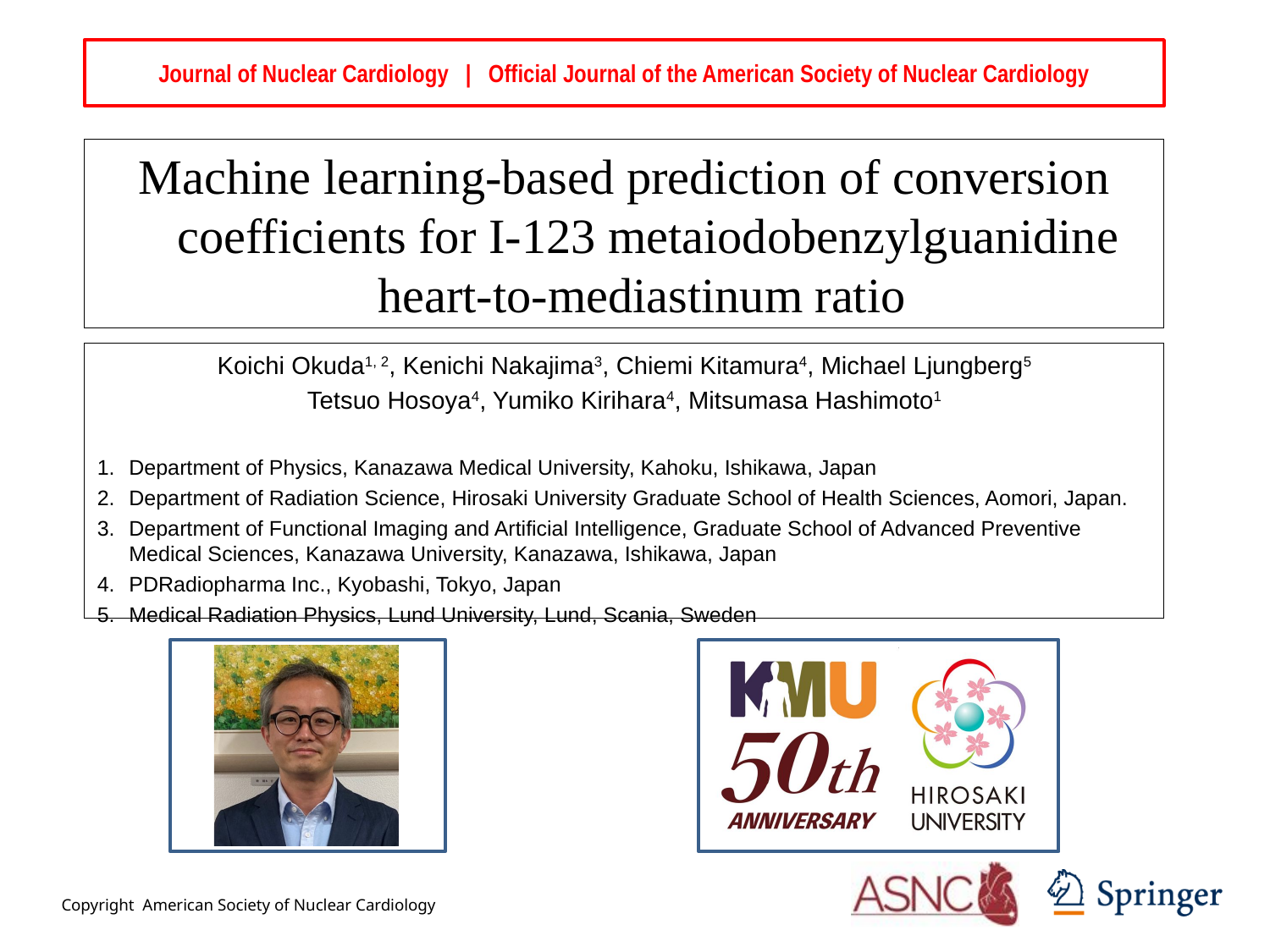

Journal of Nuclear Cardiology | Official Journal of the American Society of Nuclear Cardiology
# Machine learning-based prediction of conversion coefficients for I-123 metaiodobenzylguanidine heart-to-mediastinum ratio
Koichi Okuda1, 2, Kenichi Nakajima3, Chiemi Kitamura4, Michael Ljungberg5
Tetsuo Hosoya4, Yumiko Kirihara4, Mitsumasa Hashimoto1
Department of Physics, Kanazawa Medical University, Kahoku, Ishikawa, Japan
Department of Radiation Science, Hirosaki University Graduate School of Health Sciences, Aomori, Japan.
Department of Functional Imaging and Artificial Intelligence, Graduate School of Advanced Preventive Medical Sciences, Kanazawa University, Kanazawa, Ishikawa, Japan
PDRadiopharma Inc., Kyobashi, Tokyo, Japan
Medical Radiation Physics, Lund University, Lund, Scania, Sweden
Copyright American Society of Nuclear Cardiology

## Slide 2
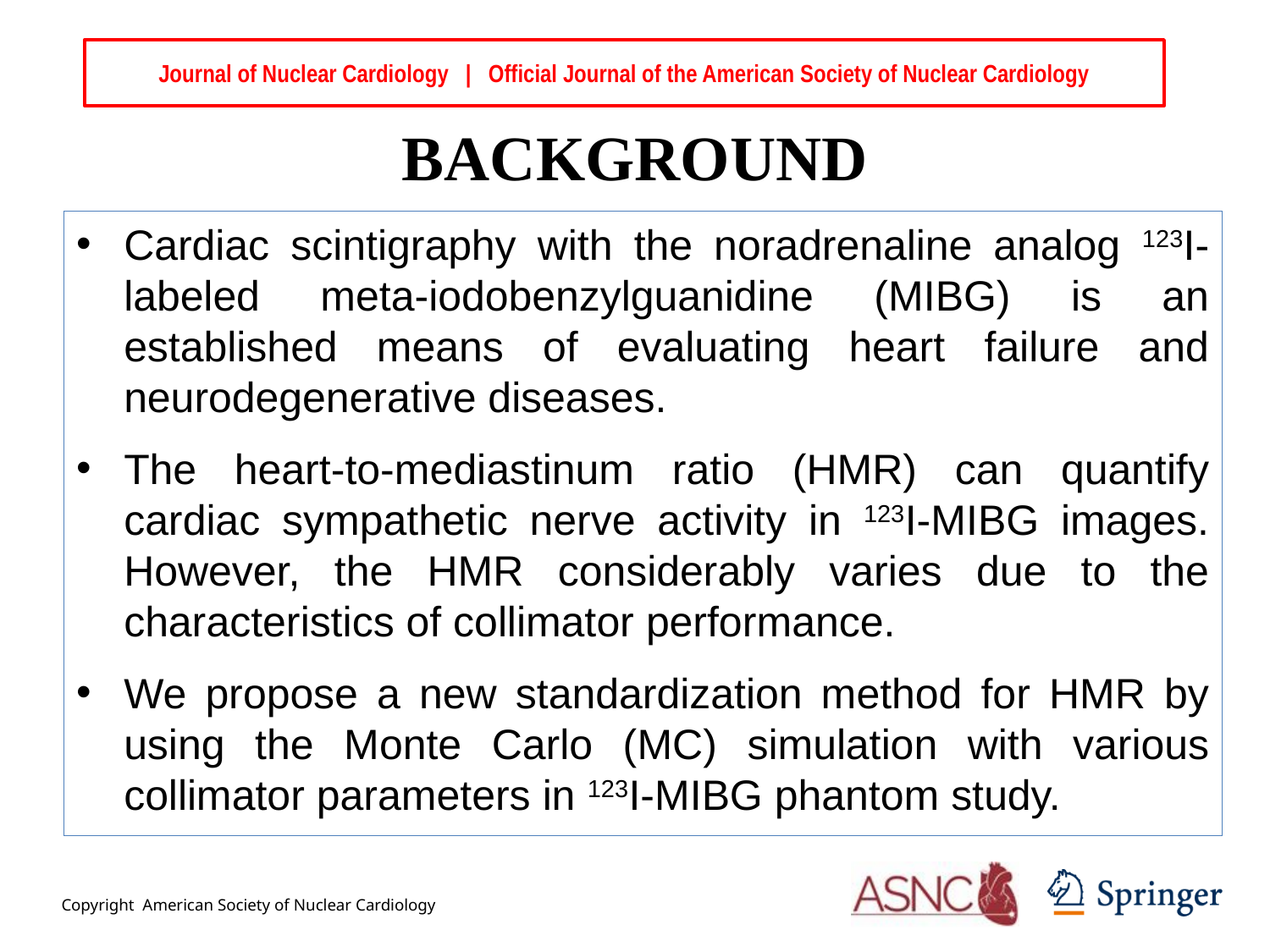

Journal of Nuclear Cardiology | Official Journal of the American Society of Nuclear Cardiology
# BACKGROUND
Cardiac scintigraphy with the noradrenaline analog 123I-labeled meta-iodobenzylguanidine (MIBG) is an established means of evaluating heart failure and neurodegenerative diseases.
The heart-to-mediastinum ratio (HMR) can quantify cardiac sympathetic nerve activity in 123I-MIBG images. However, the HMR considerably varies due to the characteristics of collimator performance.
We propose a new standardization method for HMR by using the Monte Carlo (MC) simulation with various collimator parameters in 123I-MIBG phantom study.
Copyright American Society of Nuclear Cardiology

## Slide 3
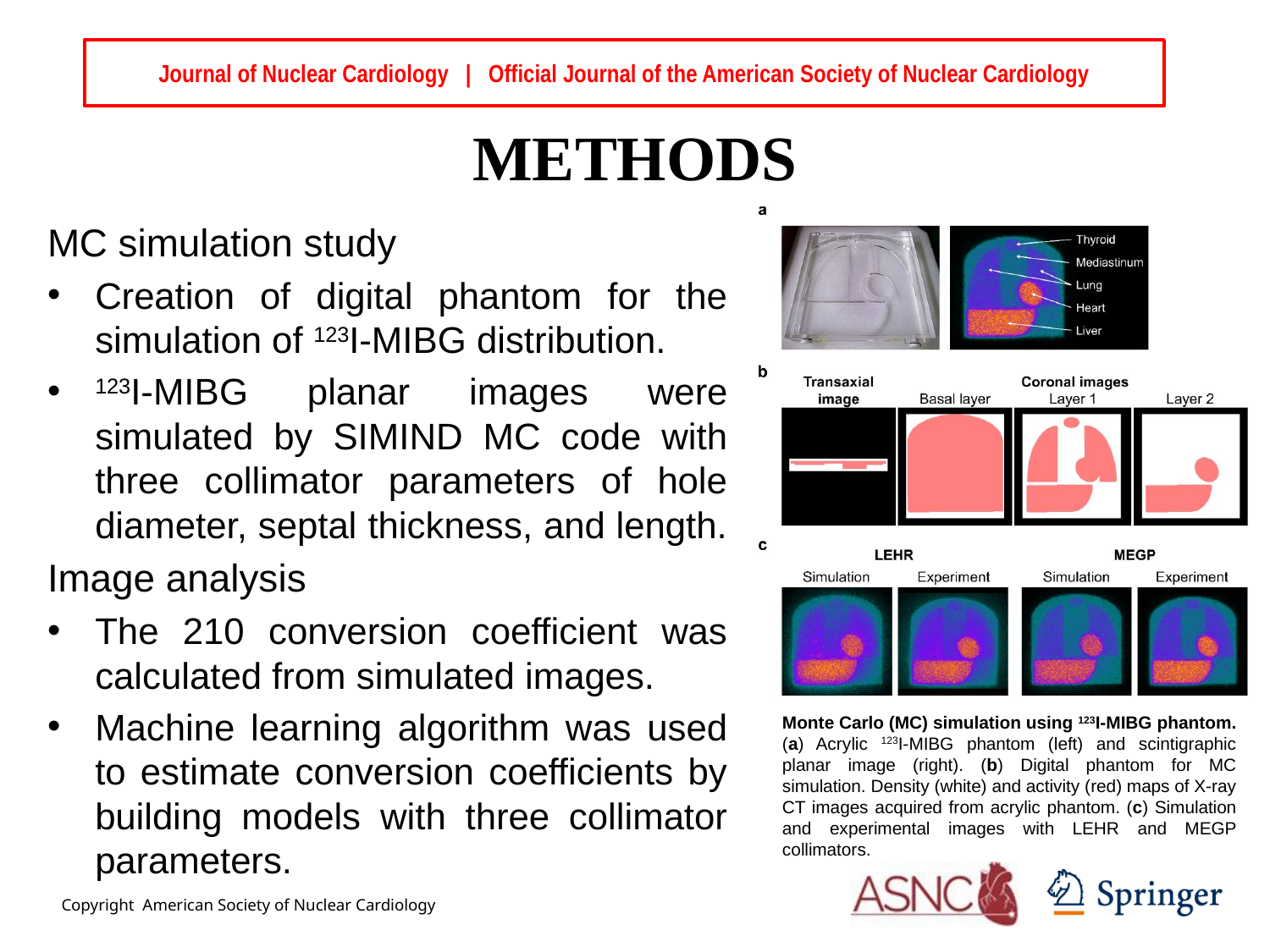

Journal of Nuclear Cardiology | Official Journal of the American Society of Nuclear Cardiology
# METHODS
MC simulation study
Creation of digital phantom for the simulation of 123I-MIBG distribution.
123I-MIBG planar images were simulated by SIMIND MC code with three collimator parameters of hole diameter, septal thickness, and length.
Image analysis
The 210 conversion coefficient was calculated from simulated images.
Machine learning algorithm was used to estimate conversion coefficients by building models with three collimator parameters.
Monte Carlo (MC) simulation using 123I-MIBG phantom. (a) Acrylic 123I-MIBG phantom (left) and scintigraphic planar image (right). (b) Digital phantom for MC simulation. Density (white) and activity (red) maps of X-ray CT images acquired from acrylic phantom. (c) Simulation and experimental images with LEHR and MEGP collimators.
Copyright American Society of Nuclear Cardiology

## Slide 4
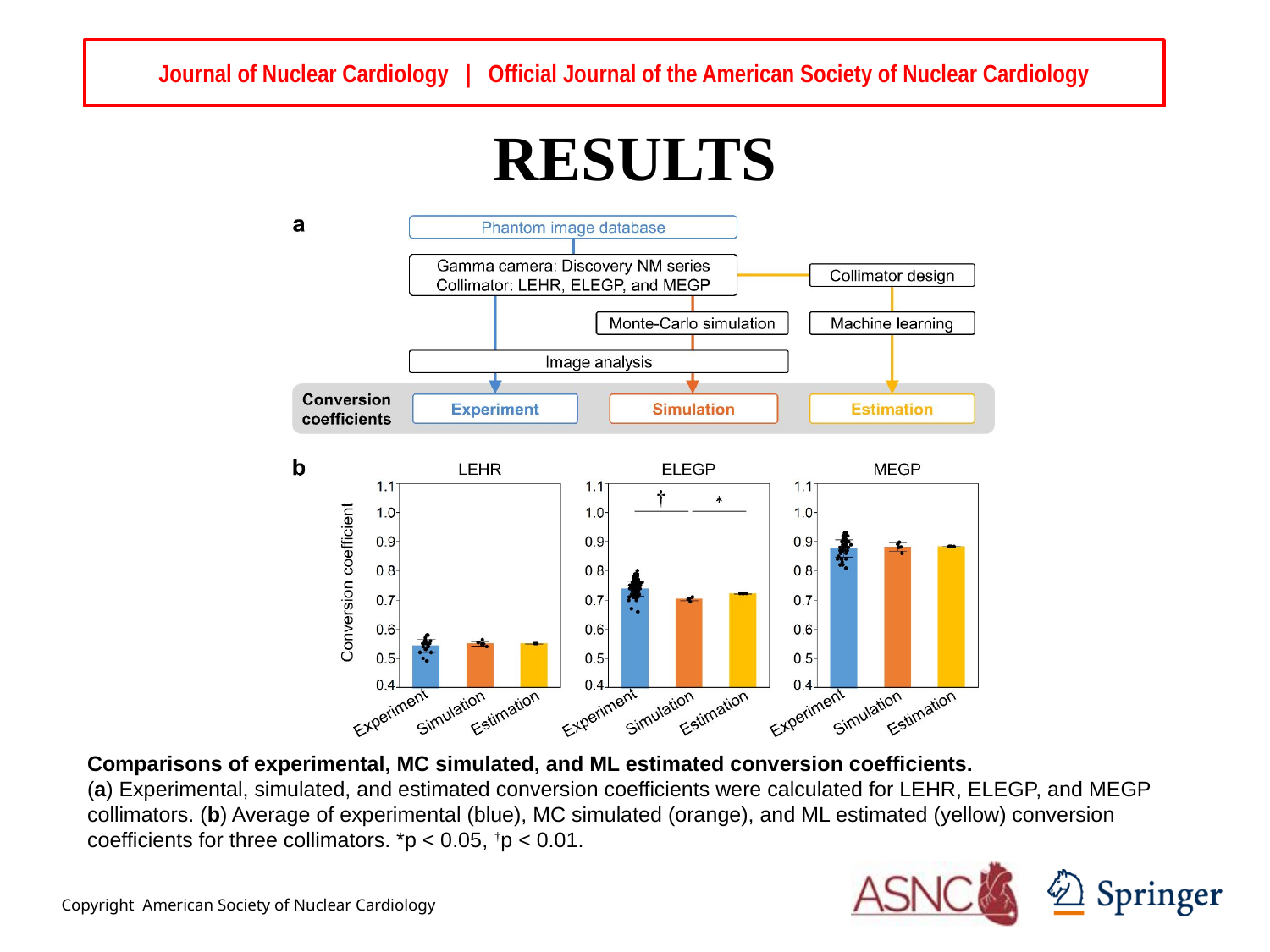

Journal of Nuclear Cardiology | Official Journal of the American Society of Nuclear Cardiology
# RESULTS
Comparisons of experimental, MC simulated, and ML estimated conversion coefficients.
(a) Experimental, simulated, and estimated conversion coefficients were calculated for LEHR, ELEGP, and MEGP collimators. (b) Average of experimental (blue), MC simulated (orange), and ML estimated (yellow) conversion coefficients for three collimators. *p < 0.05, †p < 0.01.
Copyright American Society of Nuclear Cardiology

## Slide 5
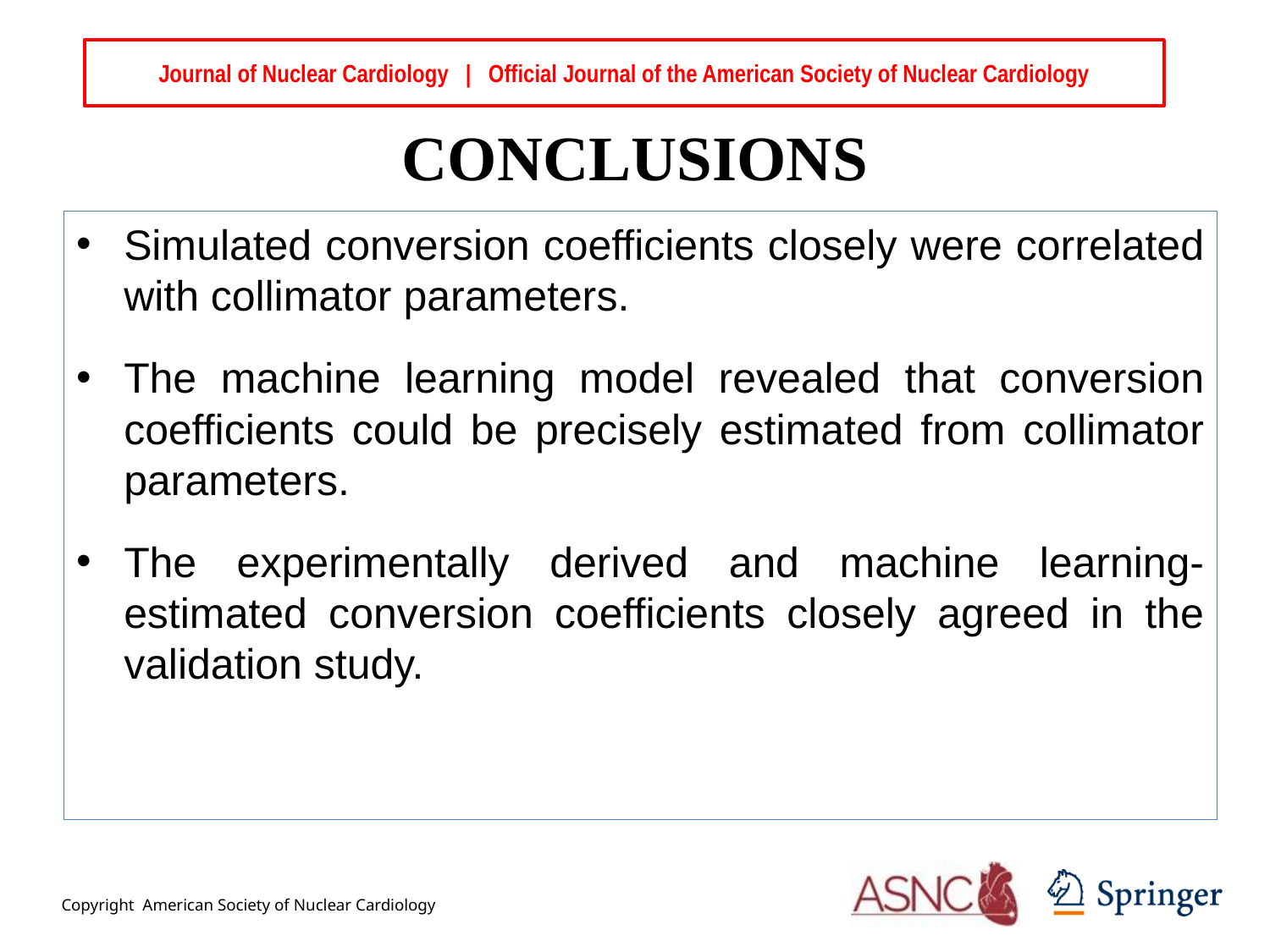

Journal of Nuclear Cardiology | Official Journal of the American Society of Nuclear Cardiology
# CONCLUSIONS
Simulated conversion coefficients closely were correlated with collimator parameters.
The machine learning model revealed that conversion coefficients could be precisely estimated from collimator parameters.
The experimentally derived and machine learning-estimated conversion coefficients closely agreed in the validation study.
Copyright American Society of Nuclear Cardiology
